# In utero nutritional stress as a cause of obesity: Altered relationship between body fat, leptin levels and caloric intake in offspring into adulthood

**DOI:** 10.1016/j.lfs.2020.117764

**Published:** 2020-05-12

**Authors:** Rogerio Sertie, Minsung Kang, Jessica P. Antipenko, Xiaobing Liu, Lidia Maianu, Kirk Habegger, W. Timothy Garvey

**Affiliations:** a Department of Nutrition Sciences, University of Alabama at Birmingham, United States of America; b Department of Medicine, University of Alabama at Birmingham, United States of America; c Birmingham Veterans Affairs Medical Center, Birmingham, AL, United States of America

**Keywords:** Obesity, Epigenetics, Maternal diet, Leptin, Metabolism

## Abstract

**Aims:**

Emerging evidence suggests that during gestation the in utero environment programs metabolism and can increase risk of obesity in adult offspring. Our aim was to study how alterations in maternal diets during gestation might alter body weight evolution, circulating leptin levels and caloric intake in offspring, leading to changes in body composition.

**Materials and methods:**

We fed gestating rats either a control diet (CD), high fat diet (HFD) or an isocaloric low protein diet (LPD), and examined the repercussions in offspring fed similar diets post-weaning on birth weight, body weight evolution, body composition, insulin sensitivity, glucose tolerance and in the relationship between plasma leptin concentration and caloric intake in offspring during growth and development.

**Key finds:**

Offspring from dams fed LPD maintained reduced body weight with greater % lean mass and consumed fewer calories despite having leptin levels similar to controls. On the other hand, offspring from dams fed a HFD were insulin resistant and maintained increased body weight and % fat mass, while consuming more calories than controls despite elevated leptin concentrations. Therefore the uterine environment, modulated primarily through maternal nutrition, modified the relationship between circulating leptin levels, body fat, and caloric intake in the offspring, and dams fed a HFD produced offspring with excess adiposity, insulin resistance, and leptin resistance into adulthood.

**Significance:**

Our data indicates that in utero environmental factors affected by maternal diet program alterations in the set point around which leptin regulates body weight in offspring into adulthood contributing to obesity.

## 1. Introduction

In 1986, Barker et al. [1] found that regions in England and Wales with high ischemic heart disease mortality in 1968–78 also had high infant mortality rates in 1921–25, relating this to the great famine that occurred during the First World War. This led to the hypothesis that nutritional stress alters the in utero environment during gestation affecting the offspring’s growth and development (Barker’s hypothesis). Subsequently, another study from the same group linked low birth weight to increased mortality from cardiovascular disease in men [2]. Since then there has been an accumulating body of evidence that offspring metabolism and body composition are prenatally programmed in utero [3–6]. Indeed, maternal diet manipulation, present before and/or during the perinatal period, represents an important form of in utero stress and may program offspring’s metabolism regardless of maternal genetics [1,2,7]. Studies in human and animal models have corroborated this theory by demonstrating a clear relationship between birth weight (high or low) with morbidities such hyperglycemia, insulin resistance, dyslipidemia and elevated body weight in offspring, whose symptoms usually appear during adulthood [8–18].

The development of obesity is induced by positive energy balance involving an excess of caloric intake and/or reduced energy expenditure [19,20]. Caloric intake is controlled by various hormones such as leptin, which binds to specific receptors (OB-Rs) located in the arcuate nucleus of the hypothalamus [21]. Alterations in satiety hormone dynamics and hypothalamic signaling can affect caloric consumption and contribute to obesity pathogenesis [22]. After released mainly by the adipocytes, leptin can cross the blood-brain barrier (BBB) giving information for the hypothalamus about the status of the body energy stores. The biological effects of leptin on hypothalamic feeding centers results in decreased caloric intake and increased energy expenditure, important factors regulating body weight [23–26]. In obesity, leptin levels are elevated and oscillate around a higher level of body weight, and the failure to suppress appetite is consistent with a state of leptin resistance [27].

While circulating satiety hormones in mothers can be transmitted to offspring through the placenta and breast milk [28,29], little is known regarding mechanisms affecting body weight and metabolism as a consequence of the in utero environment. In particular, the ability of the maternal diet to alter leptin action in offspring as a regulator of food intake is unknown. Therefore, we hypothesized that maternal dietary manipulation involving nutrient excess or isocaloric protein restriction can affect the set point around which leptin regulates food intake resulting in depleted or excessive fat accretion into adulthood. Our studies assessed the impact of maternal diet on glucose tolerance, insulin sensitivity, insulin signaling and body weight changes during growth and development, as well as the relationship between circulating leptin levels, body fat, and caloric intake in a rat model.

## 2. Material and methods

### 2.1. Rats

The UAB Institutional Animal Care and Use Committee (IACUC #20901) approved all processes and treatments. Sprague Dawley rats (cat#400) were purchased from Charles River Laboratories and maintained between 20 and 23 °C on a 12-hour day-night cycle with ad libitum food and water access. Female rats (n = 5) were randomized to a control diet (CD; 65% carbohydrate, 20% protein, 15% fat), isocaloric low protein diet (LPD; 77% carbohydrate, 8% protein, 15% fat), or high fat diet (HFD; 26% carbohydrate including sucrose, 16% protein, 58% fat). The diets were purchased from Research Diets (New Brunswick, New Jersey), and catalog numbers are D11112201, D16122703, and D17082304, respectively.

To induce metabolic disturbances in dams before pregnancy, the LPD and HFD groups were fed their respective diets for 4-weeks prior to mating with age-matched male rats, then single-housed until the delivery of pups. Within 24 h after delivery, litter-size was adjusted (n = 8) to equalize opportunities for maternal care, preserving as many males as possible. Male pups that were euthanized 24 h after birth had their blood glucose assessed using an Arkray glucometer (Minneapolis, MN). During the lactation period, dams continued to receive the same diet. Male pups were weaned at 3-weeks old and all offspring from the maternal diet subgroups were fed identical diets, randomly assigned to CD or HFD. This resulted in a total of six diet groups with 7–8 rats per group; the three offspring groups fed the CD: CD (maternal diet)-CD (offspring diet), LPD-CD, HFD-CD, and the three offspring groups fed the HFD: CD-HFD, LPD-HFD, and HFD-HFD.

### 2.2. In vivo metabolic phenotypes

Body weight and food intake measurements were assessed weekly up to 10 weeks old, during which time rats were housed individually. Body composition was measured using magnetic resonance spectroscopy (EchoMRI, Echo Medical Systems). In addition to weekly measurements, food intake was quantified on a specific day at 30 min and 1, 2, 3, 4, and 24 h after an overnight fast.

Intraperitoneal (IP) glucose tolerance tests (GTT) were performed (using 2 g/kg, 20% wt/vol d-glucose in 0.9% wt/vol saline IP, Sigma-Aldrich Corp., St. Louis, MO) in 5 h fasted rats (food was removed at 6 AM and the test was performed at 11 AM) in 4 and 9 weeks old animals. Tail vein blood glucose was assessed using an Arkray glucometer (Minneapolis, MN) at 0′, 15′, 30′, 60′ and 120′ after intraperitoneal glucose injection. Blood samples from GTTs were collected at 0, 15 and 60 min after glucose injection and plasma insulin concentrations were measured by an enzyme-linked immunosorbent assay (ELISA, Sensitive Rat insulin RIA kits, Millipore, Billerica, MA) through the Human Physiology Core of the UAB Diabetes Research Center.

### 2.3. Circulating hormone concentration

Total leptin concentrations were measured from 9 weeks old rats by ELISA (rat leptin ELISA, Millipore, Billerica, MA). Blood samples from fasting animals (12 h – 8 pm to 8 am) were collected in the same time of the day from tail veins, incubated at room temperature (RT) for 20 min, and then centrifuged at 3000 ×*g* for 15 min.

### 2.4. Insulin signaling: Immunoblot analyses

Male offspring from dams fed CD, LPD, or HFD were weaned at 3-weeks old, euthanized by decapitation with or without insulin injection (1 U/kg) and liver was extracted, homogenized in lysis buffer (20 mm Tris, pH 6.8; 3.8 mm DTT, 10% glycerol, 1% SDS and protease inhibitor cocktail [Thermo Fisher Scientific, Inc.]) and centrifuged for 10 min at 4 °C to remove the fat cake. Equal amounts of proteins (20 μg) were separated by 7.5% SDS-PAGE and transferred to PVDF membranes (Bio-Rad Laboratories, Inc., Hercules, CA). Membranes were then blocked (5% BSA in 0.1% PBST/1 h) and incubated with the following primary antibodies: phosphoAkt^473^ (1:3000) or pan-Akt^total^ (1:3000) (Cell Signaling Technology, Danvers, MA). Each membrane was subsequently incubated with goat anti-rabbit horseradish peroxidase conjugated secondary antibodies (Cell Signaling Technology, Danvers, MA). Protein bands were detected and quantified using Clarity ECL, ChemDoc imaging system, and Image Lab 5.0 software (Bio-Rad Laboratories, Inc., Hercules, CA). Phosphorylated AKT was normalized by total AKT.

### 2.5. Statistical analyses

The results are expressed as the mean ± SEM. The symmetry of the samples was tested by the Kolmogorov-Smirnov normality test. Two-way analysis of variance with Bonferroni’s post hoc test was used to compare the effects of time and treatment. The One-way ANOVA followed by post-Bonferroni tests for multiple comparisons was used to compare between the groups. All statistical tests and graphics were made using the software GraphPad Prism 6.01 for Windows, San Diego, CA, USA. Statistical significance was assigned when p < 0.05. The data were obtained by comparing the experimental groups with their respective controls, that is, the CD group is the control of the LPD and HFD groups; the CD-CD group is the control of the LPD-CD and HFD-CD groups; and the CD-HFD group is control for the LPD-HFD and HFD-HFD groups.

## 3. Results

### 3.1. Maternal diet influences phenotypes at birth

Our first step was to examine the effects of diets on body weight of dams before mating. We assessed the initial body weight, the final body weight (4 weeks after starting maternal diets, but immediately before mating), and the change in body weight over this interval (final body weight - initial body weight). There was a tendency for less weight gain in the mothers fed a LPD during the 4 weeks of treatment before mating that did not achieve statistical significance (Mothers final BW p = 0.06 CD vs LDP). On the other hand, the HFD diet did not affect the weight gain of the mothers when compared with CD mothers group (Table 1). To examine whether maternal diets can affect offspring phenotype at birth, we compared birth weights and blood glucose concentrations within 24 h after birth in the newborn rats of dams fed a CD, LPD or HFD during gestation. Offspring from dams exposed to a LPD were characterized by a 6.75% lower birth weight (p < 0.001), while offspring from dams exposed to a HFD showed the same birth weight compared to offspring from dams fed CD (Table 1). Blood glucose was also measured in these newborn animals, and offspring from dams fed LPD exhibited 18.88% higher blood glucose concentration than offspring from dams fed CD (p = 0.0098, Table 1). Offspring from dams fed HFD also showed 7.67% higher glucose concentration than offspring from dams fed CD although the difference was not quite statistically significant (p = 0.085). Given these results, our observations indicate that maternal dietary manipulation affects birth weight and blood glucose of offspring at birth.

### 3.2. Maternal diet affects systemic metabolism in offspring at weaning

**Fig. 1.**
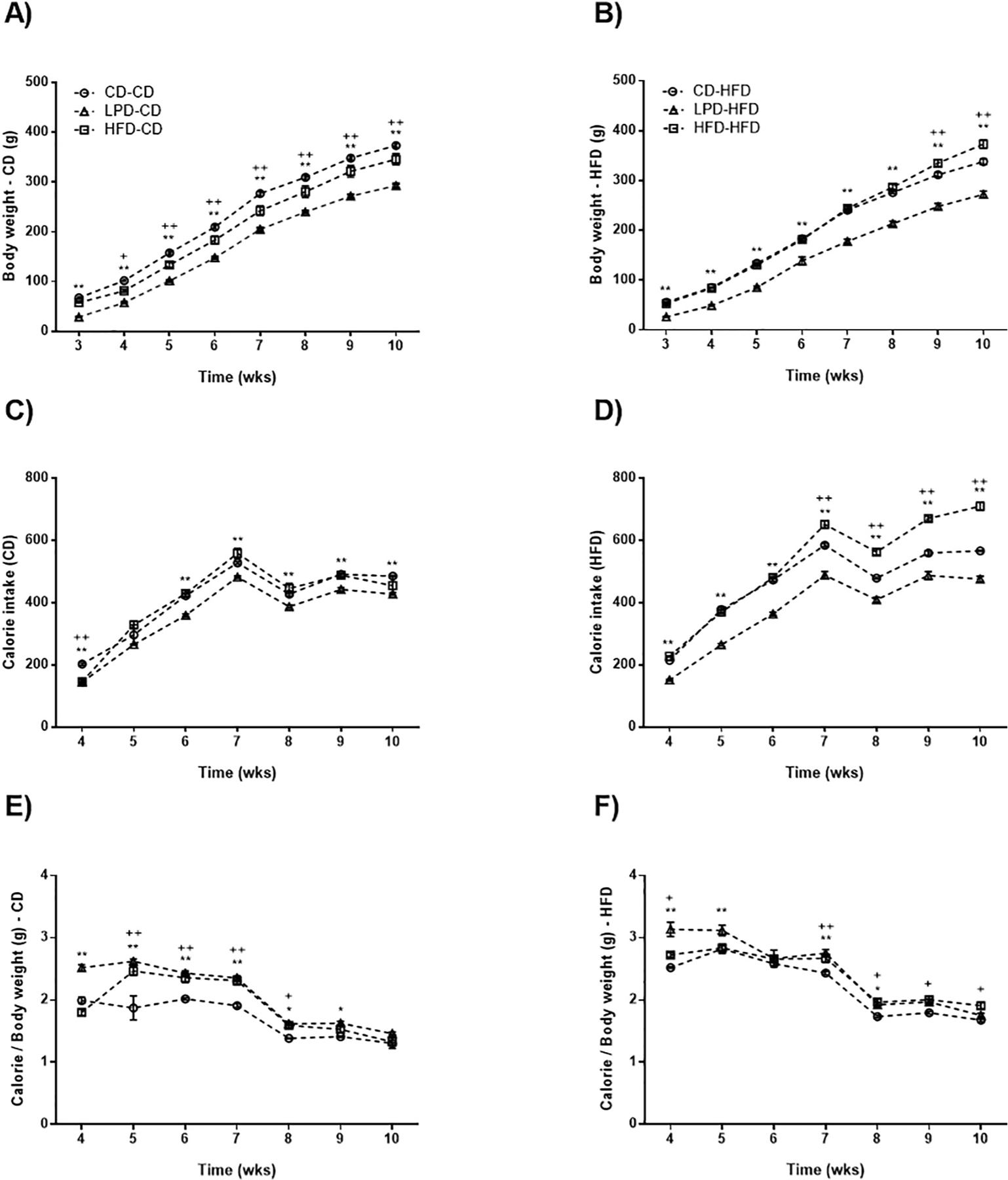
Body weight and calorie intake evolution. Male offspring from dams fed CD, LPD, or HFD were weaned at 3-weeks old and randomly assigned to CD or HFD resulting in a total of six diet groups: CD (maternal diet)-CD (offspring diet), LPD-CD, HFD-CD, CD-HFD, LPD-HFD, and HFD-HFD groups. The body weight evolution and calorie intake were followed until the animals reached 10 weeks of age. A, C and E: *p < 0,05 CD-CD vs LPD-CD; **p < 0,01 CD-CD vs LPD-CD; ^+^p < 0,05 CD-CD vs HFD-CD; ^++^p < 0,01 CD-CD vs HFD-CD. B, D and F: *p < 0,05 CD-HFD vs LPD-HFD; **p < 0,01 CD-HFD vs LPD-HFD; ^+^p < 0,05 CD-HFD vs HFD-HFD; ^++^p < 0,01 CD-HFD vs HFD-HFD. Data represents mean ± SEM, n = 7–8 each group.

**Fig. 2.**
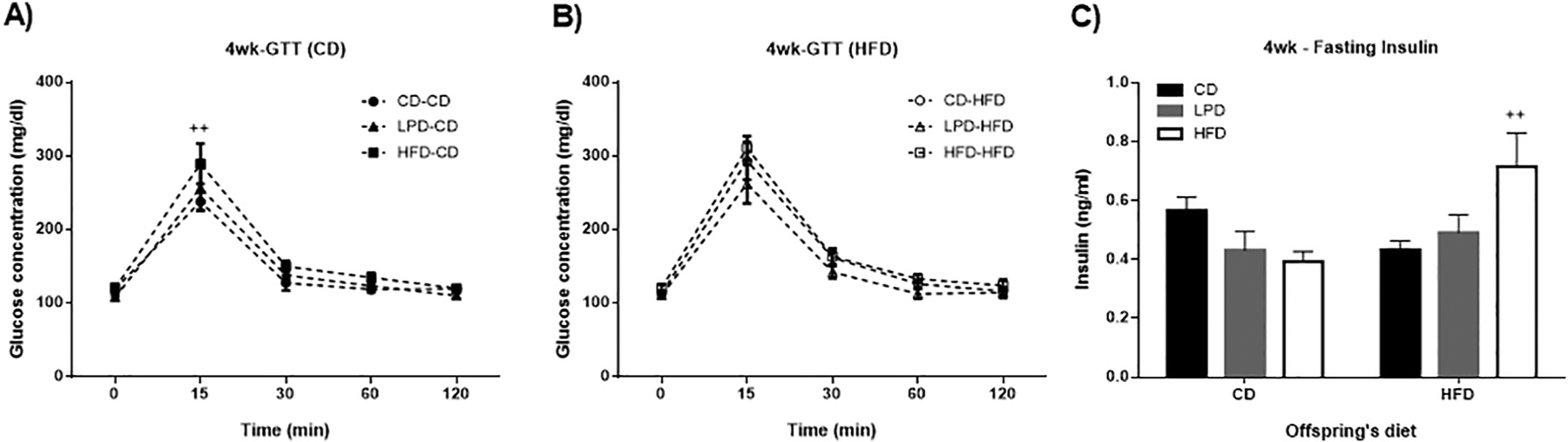
Four weeks old glucose tolerance teste and fasting insulin. Glucose tolerance test was performed one week after the offspring were exposed to CD or HFD (one week after weaning). Fasting insulin concentration was measured when offspring were at the same age (4 weeks old). A: 4 weeks old glucose tolerance test in offspring who received CD after weaning - ^++^p < 0,01 CD-CD vs HFD-CD. B: 4 weeks old glucose tolerance test in offspring who received HFD after weaning. C: 4 weeks old fasting insulin - ^++^p < 0,01 CD-HFD vs HFD-HFD. Data represent mean ± SEM, n = 7–8 per group.

**Fig. 3.**
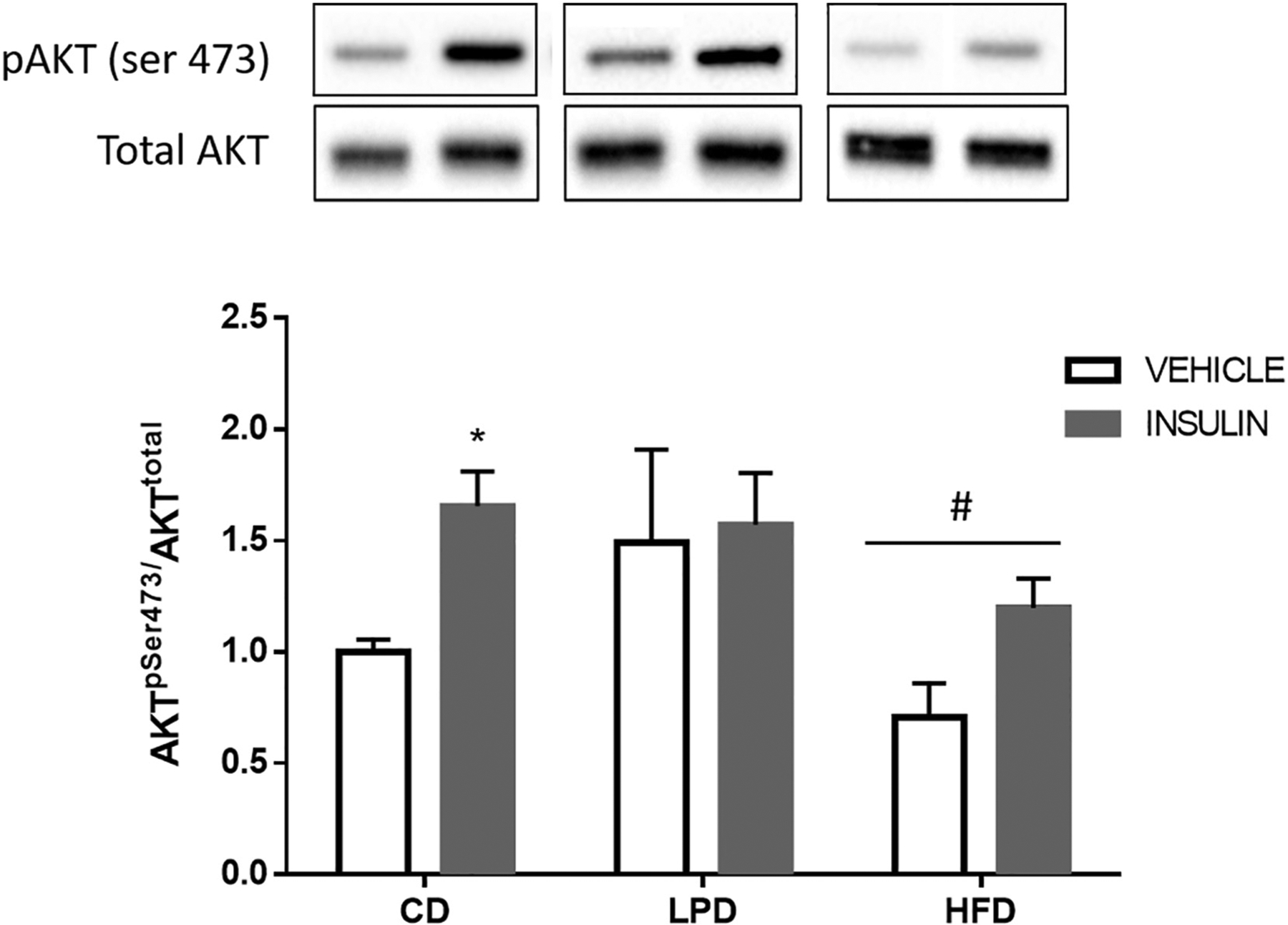
Maternal dietary manipulation effect on hepatic AKT phosphorylation. Male offspring from dams fed CD, LPD, or HFD were weaned at 3-weeks of age and liver was dissected with or without insulin injection (1 U/kg) prior euthanasia. Liver samples of each group were processed and the proteins quantified by western blot. A: representative blots of protein levels of phosphorylated AKT and total AKT. B: western blotting results were quantified by Image Lab, and total AKT was used for normalization. *p < 0.05 significant difference between basal CD and insulin-stimulated CD; ^#^p < 0.05 significant difference between CD group vs HFD group. Data represent mean ± SEM, n = 4–6 per group.

The relative differences in body weights observed at birth persisted until the time of weaning at 3 weeks of age (Table 1, Fig. 1A and B). After body weights were measured, the offspring within each litter were randomly assigned to ad libitum CD or HFD for further growth and development, and GTTs were performed one week later after beginning these new diets (4 weeks old). CD-fed offspring from dams fed HFD (HFD-CD) exhibited a 30.05% elevation in blood glucose at 15 min after glucose injection compared to CD-fed offspring of dams fed the CD (CD-CD) or LPD (LPD-CD) (Fig. 2A). When the offspring were fed a HFD, glucose tolerance in 4 weeks old offspring was similar in all groups regardless of maternal diet (Fig. 2B); however, importantly, HFD-HFD group exhibited 65.47% higher fasting insulin levels than the CD-HFD group (p = 0.013; Fig. 2C). The elevated insulin in the HFD-HFD group was suggestive of insulin resistance. To examine whether the HFD in dams could alter insulin signaling in offspring, we assessed insulin’s ability to induce Akt phosphorylation in liver from offspring at weaning (3 weeks of age). We observed that offspring from dams fed with CD showed a clear increment in insulin-stimulated AKT phosphorylation above baseline (Fig. 3). In contrast, LPD and HFD groups lost the ability to respond to insulin stimulation (no difference between baseline and insulin-stimulated state). Even so, total immunoreactive phospho-AKT was lower under both basal and insulin stimulated conditions in offspring from HFD mothers compared with the corresponding values from CD and LPD groups. (^#^p < 0.05 significant difference between CD group vs HFD group, Fig. 3). These results indicate that in utero exposure to maternal high-fat feeding impairs insulin action in offspring.

### 3.3. Maternal diets affects offspring body weight and composition during growth and development

Maternal diet affected the body weight of offspring after weaning and into adulthood despite the fact that offspring groups were allowed ad libitum access to the same diet. Body weight was measured weekly for a 10-week period following weaning while offspring were fed either the CD or HFD, as shown in Fig. 1. Offspring from all 3 maternal diet subgroups progressively gained weight. However, among offspring fed CD after weaning (Fig. 1A), the group LPD-CD exhibited lower body weight compared to CD-CD group (statistically significant between 3 and 10-weeks old). Similarly, HFD-CD exhibited lower body weight evolution (Fig. 1A) than CD-CD animals (statistically significant between 4 and 10-weeks old) with values intermediate between CD-CD and LPD-CD. In rats fed HFD after weaning (Fig. 1B), rats from LPD-fed mothers exhibited reduced body weight at all time points relative to those from CD and HFD fed dams. The offspring from HFD-fed mothers showed an interesting pattern. Body weights from 3 to 7 weeks were similar in HFD-HFD and CD-HFD animals, but at 7 weeks weight gain in HFD-HFD rats accelerated and rose above weights observed in CD-HFD rats at 9 and 10 week time points (Fig. 1B).

Body composition in all offspring subgroups (Table 1) was at 10-weeks of age. Lower body weight in offspring of dams fed a LPD was associated with both lower lean mass (p < 0.0001 CD-CD vs LPD-CD) and lower fat mass (p < 0.001 CD-CD vs LPD-CD) compared with corresponding controls. When normalized to total body weight, LPD offspring exhibited reduced % fat (p < 0.05 CD-CD vs LPD-CD) but similar % lean mass (p = 0.784) compared with controls (CD-CD). On the other hand, the higher body weight in HFD-HFD group was due to increments in both fat and lean mass (both p < 0.01); however, when normalized to body weight, the HFD-HFD rats had greater % fat (p < 0.01) and similar % lean mass (p = 0.15) compared with CD-HFD animals. Together these data indicate that maternal diets affect both body weight and body composition in older offspring.

### 3.4. Maternal diet influences Offspring’s food consumption

Previous reports suggest that maternal obesity can alter caloric intake in adult offspring [12–14]. To assess the effects of our maternal diets on caloric intake, we measured the weekly calorie intake in each offspring experimental group (Fig. 1C, D, E, F). In utero exposure to maternal protein restriction suppressed food intake in both LPD-CD and LPD-HFD rats as compared to their diet matched controls (CD-CD and CD-HFD, respectively). Conversely, in utero exposure to a HFD resulted in general higher food intake in both HFD-CD and HFD-HFD as compared to their respective controls (CD-CD and CD-HFD, respectively).

**Fig. 4.**
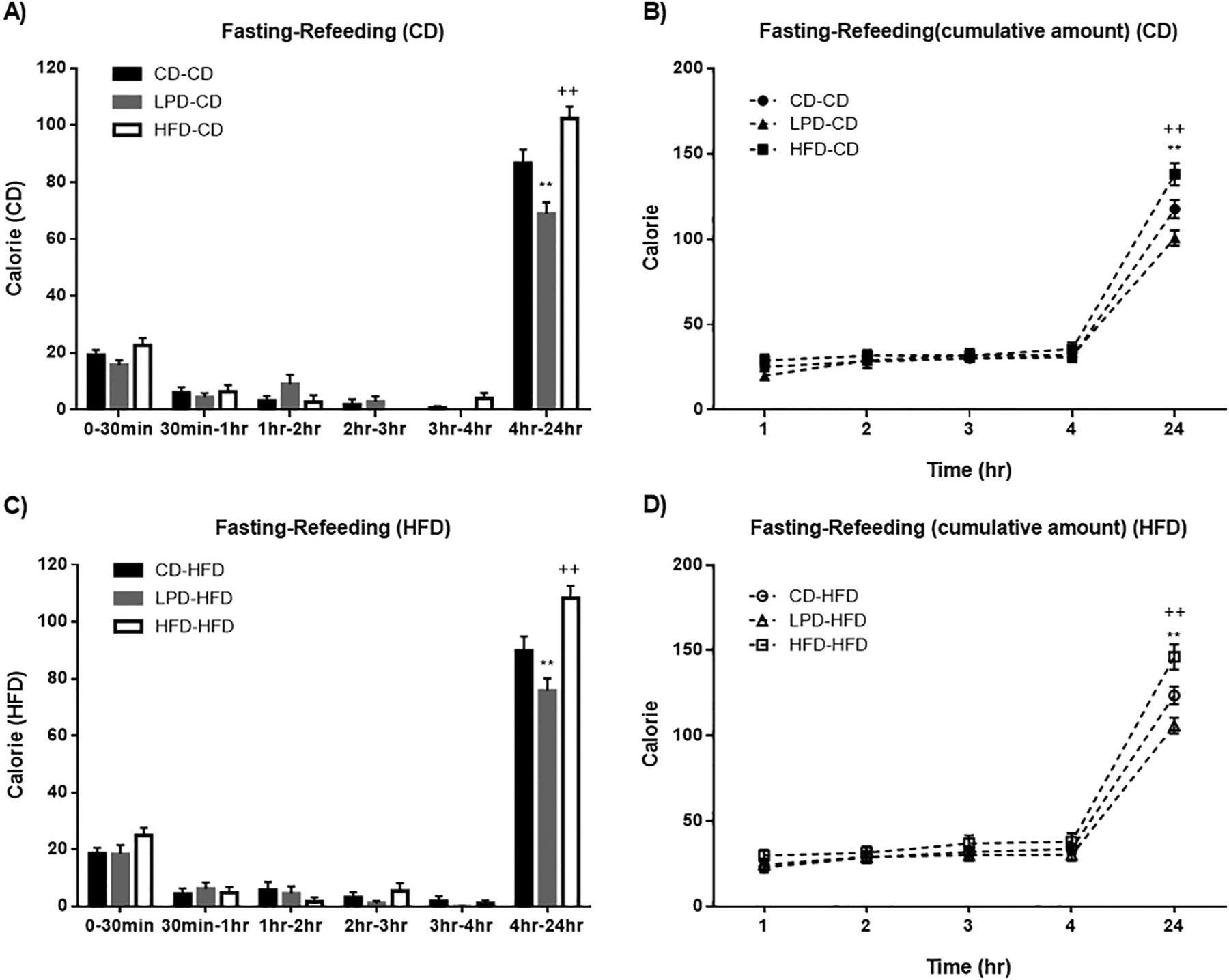
Maternal dietary manipulation effect on fasting-refeeding test. Male offspring from dams fed different diets (CD, LPD or HFD) were weaned at 3-weeks old and randomly assigned into CD or HFD diets. A: fasting-refeeding test on offspring fed with CD after weaning (expressed in calories) - ^++^p < 0,01 CD-CD vs HFD-CD; **p < 0,01 CD-CD vs LPD-CD. B: fasting-refeeding test on offspring fed with CD after weaning (expressed in cumulative amount of calorie) - ^++^p < 0,01 CD-CD vs HFD-CD; **p < 0,01 CD-CD vs LPD-CD. C: fasting-refeeding test on offspring fed with HFD after weaning (expressed in calories) - ^++^p < 0,01 CD-HFD vs HFD-HFD; **p < 0,01 CD-HFD vs LPD-HFD. D: fasting-refeeding test on offspring fed with HFD after weaning (expressed in cumulative amount of calorie) - ^++^p < 0,01 CD-HFD vs HFD-HFD; **p < 0,01 CD-HFD vs LPD-HFD. Data represent mean ± SEM, n = 7–8 per group.

To examine whether the effect of maternal diet to alter food intake in adult offspring was associated with fasting duration, we next assessed ad libitum caloric intake over a 24 h period following an overnight fast (Fig. 4). Regardless of whether consuming a CD or HFD diet, offspring from HFD-fed dams displayed trends for greater calorie intake as compared to offspring from CD- and LPD-fed dams during the first 30 min (Fig. 4A and C); however, differences in caloric intake were minimal and not consistent when integrated over the first 4 h. It was over the period of 4–24 h that differences in caloric intake became clearly evident. Over this time interval, offspring of LPD-fed dams consumed significantly less than offspring of CD-fed dams and offspring of HFD-fed dams consumed more calories than offspring of CD-fed dams. Thus, when food was made available following an overnight fast, maternal diets had a more profound effect to on the delayed feeding response in offspring from 4 to 24 h but not the early feeding response (0–4 h).

### 3.5. Maternal diet influences relationship between leptin concentrations and body weight in offspring

**Fig. 5.**
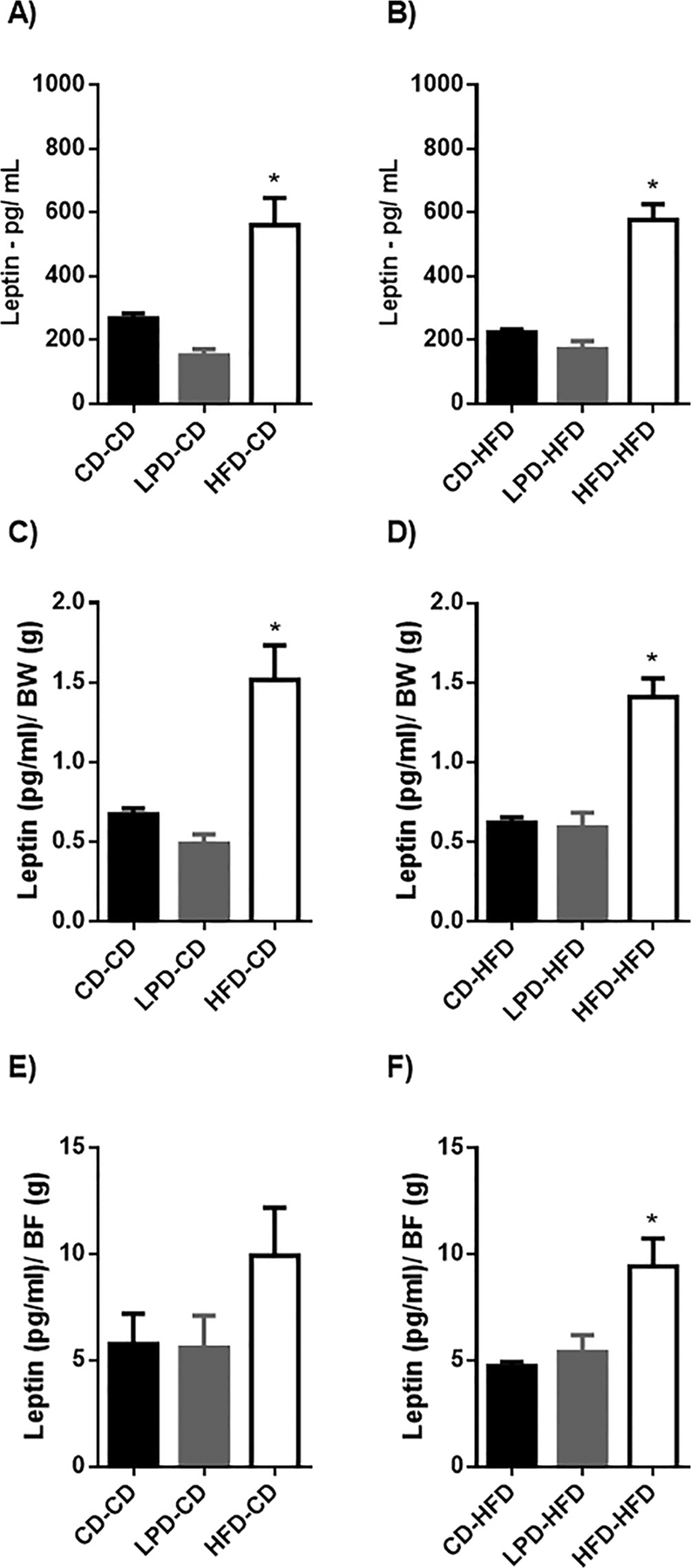
Measurement of leptin concentration. Circulating leptin concentration after an overnight fast (12 h) was measured in 9 weeks old male offspring of dams fed CD, LPD or HFD. A: circulating leptin level in offspring fed with CD after weaning (pg/mL) - *p < 0,05 CD-CD vs HFD-CD. B: circulating leptin level in offspring fed with HFD after weaning (pg/mL) - *p < 0,05 CD-HFD vs HFD-HFD. C: circulating leptin level in offspring fed with CD after weaning ((pg/mL)/BodyWeight) - *p < 0,05 CD-CD vs HFD-CD. D: circulating leptin level in offspring fed with HFD after weaning ((pg/mL)/BodyWeight) - *p < 0,05 CD-HFD vs HFD-HFD. E: circulating leptin level in offspring fed with CD after weaning ((pg/mL)/BodyFat). F: circulating leptin level in offspring fed with HFD after weaning ((pg/mL)/BodyFat) - *p < 0,05 CD-HFD vs HFD-HFD. Data represent mean ± SEM, n = 7–8 per group.

We measured circulating leptin concentrations in fasting rats to determine whether this important satiety hormone could explain the differences in caloric intake and body weight among our offspring subgroups. Fig. 5A and B shows leptin levels in 10-week old rats. In offspring of dams fed LPD, leptin levels were similar to those observed in offspring from CD-fed dams, whether offspring were maintained on CD or HFD, despite having lower caloric intake and reduced body fat. When gestational rats were fed HFD, leptin levels in offspring were increased compared to controls on either CD or HFD despite having increased caloric intake and body fat. Thus, the data reflected differences in leptin sensitivity regarding the impact of maternal diet on caloric intake. Furthermore, maternal diet had altered the set point around which leptin regulated food intake, fat accretion, and body composition. To further examine this issue, leptin levels were normalized by body weight (BW - Fig. 5C and D) and body fat (BF – Fig. 5E and F), and the relationships among the subgroups held constant.

### 3.6. Maternal diet influences glucose homeostasis in offspring

**Fig. 6.**
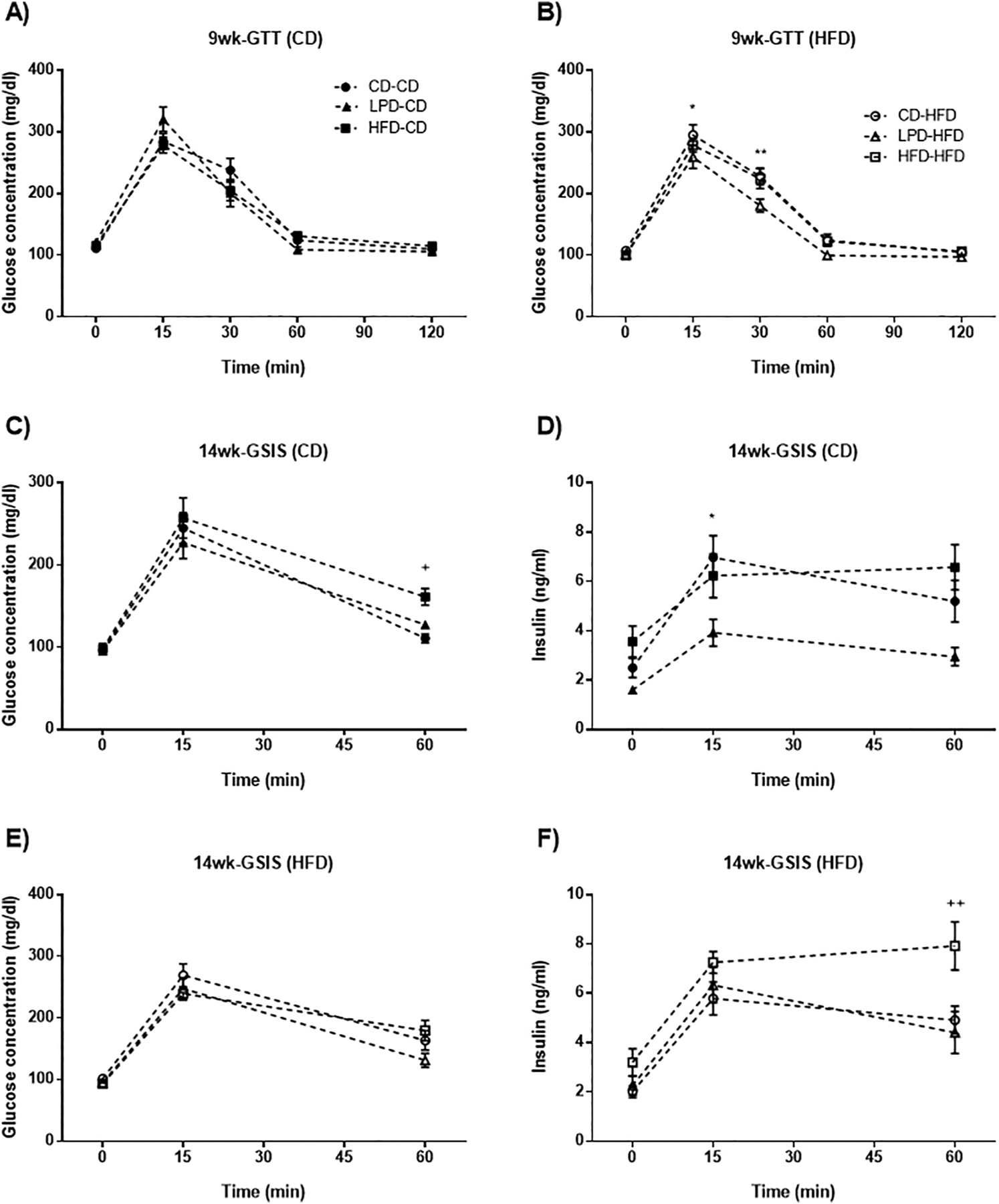
Offspring exhibit different metabolic phenotypes depending on dam’s diets in adulthood. A: 9 weeks old GTT in male offspring fed with CD after weaning (mg/dL). B: 9 weeks old GTT in male offspring fed with HFD after weaning (mg/dL) - *p < 0.05, **p < 0.01 significant difference between CD-HFD and LPD-HFD groups. C and D: GSIS test in 14-weeks old offspring fed with CD after weaning - ^+^p < 0.05 CD-CD vs HFD-CD; *p < 0.05 CD-CD vs LPD-CD. E and F: GSIS test in 14-weeks old offspring fed with HFD after weaning - ^++^p < 0,01 CD-HFD vs HFD-HFD. Data represent mean ± SEM, n = 7–8 per group.

We next investigated the effects on adult glucose homeostasis at different stages of development. GTTs were repeated at 9 and 14 weeks of age. At the age of 9 weeks, when rats are considered young adults [30], there was no effect of maternal diet on glucose tolerance among offspring groups fed a CD diet (Fig. 6A). However, among HFD-fed rats, the LPD-HFD group exhibited reduced blood glucose at 15 and 30 min as compared to the CD-HFD rats (p = 0.039, Fig. 6B), but no difference was observed in the comparison between CD-HFD vs HFD-HFD. When the rats achieved an age of 14 weeks, both glucose and insulin levels (GSIS) were assessed during the GTTs (Fig. 6C, D, E and F). In offspring from LPD-fed mothers fed a CD (LPD-CD), glucose levels during the GTT were similar to CD-CD controls (Fig. 6C) despite having substantially reduced insulin levels (Fig. 6D), consistent with greater insulin sensitivity in the LPD-CD subgroup. When these offspring were fed a HFD, glucose (Fig. 6E) and insulin levels (Fig. 6F) in LPD-HFD were similar to the CD-HFD controls suggesting that the relative state of enhanced insulin sensitivity was now mitigated by HFD feeding. In offspring from HFD-fed mothers, the HFD-CD rats displayed a higher glucose level at 60 min and higher insulin at the 15 min time point compared with CD-CD controls (Fig. 6C and D) While being fed a HFD, the HFD-HFD offspring had similar glucose levels compared with CD-HFD but higher insulin at 60 min. While these differences are not dramatic, the data suggest a mild state of relative insulin resistance in offspring from HFD dams.

## 4. Discussion

Obesity and overweight are very common affecting 35% of the world population [31–34], and are directly related to the development of heart disease, stroke, diabetes, cancer, metabolic syndrome, hypertension, and increased mortality [35]. While behavioral and genetic factors are often emphasized as causal factors of this disease, much less is known about the influence of the utero environment on the genesis of obesity. In the current study, we altered the in utero environment by manipulating diet in gestational rats. Two maternal diets were tested. An isocaloric protein-restricted diet (LPD) was intended to create nutritional stress while dams were allowed ad libitum access to food. Secondly, a caloric-dense and palatable western diet (HFD) was employed as a model of nutrient excess. Our data show that the in utero environment, as influenced by maternal diet, affected body weight and body composition, leptin levels, glucose tolerance, systemic insulin sensitivity, and hepatic insulin signaling in offspring. Importantly, maternal dietary manipulation altered the relationship between circulating leptin levels, body fat, and caloric intake, and altered the set point around which leptin regulates fat mass.

Offspring from LPD-fed dams displayed reduced body weight compared with controls at birth and throughout development into early adulthood, despite having ad lib access to either regular chow or a HFD. The reduction in body weight asymmetrically affected muscle and fat and was characterized by an increase in % lean and a decrease in % fat in body composition studies. Caloric ingestion was reduced compared with controls despite the fact that leptin levels were similar to controls, which in isolation would have been expected to. Different experimental models that involve a reduced supply of nutrients to the fetus have previously been shown to result in low birth weight, including low protein diet (LPD), maternal food restriction, and uterine artery ligature (UAL) [36–45]. In our study, the LPD-fed dams were not restricted with respect to calories but consumed a diet containing ~60% less protein by weight.

Newborns from HFD-fed dams showed no alterations in birth weight when compared to the control group; however, in adulthood, these animals showed an increase in total fat and % body fat mass, together with reduced % lean mass, regardless of diet (CD or HFD after weaning). Caloric intake in offspring from HDF-fed mothers was increased despite having higher leptin levels, which would be predicted to decrease appetite.

On balance, our data indicate that nutritional stress during gestation alters the relationship between caloric intake, leptin levels, and body fat in adult offspring. Specifically, leptin levels fluctuate around a lower % body fat in offspring of LPD-fed mothers, and a higher % body fat in HFD-fed dams. Increased energy intake is a primary driver of sustained positive energy balance and weight gain (3). Although elevated leptin concentrations reflect excess energy availability and the need to reduce energy intake, a state of relative leptin resistance arises in obesity [46,47]. Our studies indicate that the dams fed with HFD produce offspring that are leptin resistant, since increased calorie intake is maintained despite elevated plasma leptin. Our data are consistent with previous studies showing that chronically obese dams (i.e., chronic obesity that preceded gestation) have offspring that exhibit higher serum leptin concentrations and decreased leptin sensitivity compared with those born of lean dams [9,11,13,14].

Leptin levels rise with overfeeding and act to restore energy balance via anorexigenic effects on feeding centers in the hypothalamus and by increasing of energy expenditure [48]. Leptin action involves interaction with membrane receptors (OB-Rs), followed by activation of signaling pathways involving the proteins JAK (Janus kinase) and STAT (Signal transducers and activators of transcription) [49,50]. The receptor is expressed in the hypothalamus and in several peripheral sites including skeletal muscles, adipose tissue, heart, adrenal glands, kidneys, immune cells, liver and pancreas [51]. In addition to the anorexigenic action, Leptin is directly involved in the regulation of adipose tissue metabolism, inhibiting lipogenesis and stimulating lipolysis and glucose oxidation [52]. In skeletal muscle it activates AMP-activated protein kinase (AMPK), directly or indirectly through mediated responses through the hypothalamus, increasing, in both cases, the oxidation of fatty acids and the uptake of glucose [53–57].

Leptin resistance may arise due to defects in hormone signaling, at the level of the hormone-receptor complex or involving intracellular signaling pathways. For example, leptin, acting via JAK2/STAT3, induces the formation of SOCS3 that feeds back to inhibit leptin signaling, leading to disruption in signal propagation. Any malfunction in this intracellular negative feedback mechanism may result in increased caloric intake and reduced energy expenditure even in the face of elevated plasma leptin levels. Leptin resistance could block the normal actions of hyperleptinemia, whether due to overfeeding or continuous subcutaneous leptin infusion, to inhibit feeding, increase energy expenditure, and reduce body weight.

There are other hormones that control food intake and body weight including anorexigenic hormones like glucagon-like peptide-1 (GLP-1) and peptide YY (PYY) as well as orexigenic hormones such as ghrelin. Of course the in utero environment could be affecting the actions of these other factors in addition to leptin [29,58,59]. For example, one study showed that PYY and GLP-1 levels in plasma were higher in offspring from dams fed a high fiber diet compared with dams fed control or high protein diets [60]. These studies underscore the need for further research to assess the contributions of the entire panel of satiety hormones and hypothalamic signaling in the intergenerational transmission of obesity.

One potential mechanism underlying in utero programming of offspring metabolism is DNA methylation, which can arise in utero and alter gene expression without modifications in nucleotide sequences [61]. For example, emerging data suggest that the POMC promoter, which is critical for hypothalamic leptin signaling, is highly methylated in obesity [62]. Hypermethylation of gene promoter regions are associated with suppression of gene expression and these modifications, along with leptin resistance and obesity, can be inherited and passed to subsequent generations [61,62]. Additionally, the methylation of the leptin gene has been observed in different models of animal obesity, reinforcing the idea of the association between leptin methylation and the development of obesity [63–65]. In the disease of obesity, the “set point” or “settling point” around which leptin and other satiety hormones determine an equilibrium body weight is adjusted upwards to maintain a higher adipose tissue mass [66], and we are suggesting that this “set point” can be modified through epigenetic responses whose origin is the maternal diet during pregnancy.

Positive energy balance can be also induced through a reduction in energy expenditure, which can be influenced by the amount of lean body mass [67]. We found that maternal dietary manipulation regulated offspring lean mass. Offspring of mothers fed LPD were found to have both lower body weight and increased % lean mass. Conversely, both HFD-CD and HFD-HFD rats showed significant reduction in % of lean mass compared to their CD counterpoints (CD-CD and CD-HFD). These results are consistent with previous studies suggesting that gestational events can influence lean mass in offspring and can help explain individual variation in energy expenditure [68,69]. In skeletal muscle, increases in the function or quantity of mitochondria augment energy expenditure and have been associated with reduced obesity risk [70–72]. Although we did not measure mitochondrial function in the current project, previous studies found that offspring of dams fed a HFD had decreased protein expression and activity of mitochondrial complexes when compared to offspring of dams fed a normal chow diet [13,15,17]. The origin of dysfunctional mitochondria in offspring could be ascribed to epigenetic modifications arising in utero, or, alternatively, impaired mitochondria could have derived from the oocytes of dams [73,74]. Saben and colleagues found that impaired mitochondria were passed on from dams to offspring for three generations through the transmission in oocytes [17].

Maternal diets also affected glucose homeostasis and insulin sensitivity in adult offspring. At 14 weeks old, glucose tolerance during the GTT in the LPD-CD subgroup was similar to that in CD-CD, while insulin levels were substantially reduced, indicative of enhanced insulin sensitivity. This phenomenon may be related to the increase in the percentage of lean mass observed in the LPD-CD animals. On the other hand, the GTT glucose and insulin values in the offspring of HFD mothers suggested a state of mild insulin resistance. This phenotype was supported by molecular changes in the liver, since offspring from HFD fed dams showed reduced ability to promote AKT phosphorylation in response to insulin. Our findings are consistent with previous studies showing that offspring from chronically obese dams exhibited impaired glucose tolerance associated with impaired insulin action in metabolically important tissues such as liver and skeletal muscle [9,11].

## 5. Conclusion

In summary, when compared to offspring of mothers fed a CD, we found that offspring from gestational rats dams fed LPD maintained reduced body weight with lower % fat and greater % lean mass, and consumed fewer calories despite having leptin levels similar to the controls. On the other hand, offspring from dams fed a HFD were relatively insulin resistant and maintained increased body weight and % fat mass, while consuming more calories than controls despite having elevated leptin concentrations. Thus, our findings strongly suggest that the uterine environment, modulated by maternal diet, modifies the relationship between circulating leptin levels, body fat, and caloric intake in the offspring. The data allows us to infer that alterations in the set point (or settling point) around which leptin regulates body weight could arise through processes operative in utero. In particular, HFD during pregnancy creates a scenario highly favorable to the fat mass gain and development of obesity in adulthood. Overall, maternal diets and the in utero environment can contribute to the intergenerational transmission of obesity.

## Figures and Tables

**Table 1:** Maternal dietary manipulation effects on body weight, glucose concentration and body composition of offspring.

	CD	LPD	HFD	CD-CD	LPD-CD	HFD-CD	CD-HFD	LPD-HFD	HFD-HFD
Mothers initial BW	171.59 ± 1.36	166.8 ± 3.97	166.5 ± 3.54	-	-	-	-	-	-
Mothers final BW	237.9 ± 3.8	218.7 ± 7.17	239.3 ± 5.26	-	-	-	-	-	-
Mothers Δ BW	66.31 ± 4.39	51.96 ± 4.75	72.8 ± 7.25	-	-	-	-	-	-
Birth weight (g)	6.96 ± 0.14	6.15 ± 0.11*	6.66 ± 0.14	-	-	-	-	-	-
Birth glucose (mg/dL)	52.39 ± 1.86	61.26 ± 3.0*	55.48 ±1.2	-	-	-	-	-	-
BW at weaning (g)	61.55 ± 1.8	27.5 ± 0.8*	55.34 ± 0.9	-	-	-	-	-	-
BW at 10 wks old (g)	-	-	-	395.3 ± 3.61	308.7 ± 4.8*	368.1 ± 11.18**	360.2 ± 4.96	291.8 ± 7.0^#^	405.3 ± 9.2^##^
QMR - fat (g)	-	-	-	39.19 ± 0.89	23.47 ± 2.2*	49.15 ± 2.0**	46.31 ± 1.57	31.78 ± 3.18	71.49 ± 7.0^##^
QMR - fat (%)	-	-	-	9.91 ± 0.19	7.6 ± 0.8*	13.32 ± 0.32**	12.87 ± 0.55	10.83 ± 0.95	17.6 ± 1.48^##^
QMR - lean (g)	-	-	-	346.2 ± 3.0	275.3 ± 7.08*	307.3 ± 9.55**	303.4 ± 5.62	250.3 ± 5.64^#^	323.5 ± 7.47
QMR - lean (%)	-	-	-	87.6 ± 0.18	89.13 ± 0.97	83.47 ± 0.24**	84.2 ± 0.59	85.82 ± 0.99	79.88 ± 1.49
QMR - water (g)	-	-	-	278.2 ± 2.18	221.7 ± 5.4*	248.4 ± 7.54**	245.2 ± 4.17	203.6 ± 4.69^#^	260.9 ± 5.88
QMR - water (%)	-	-	-	70.41 ± 0.28	71.78 ± 0.11	67.5 ± 0.34**	68.05 ± 0.43	69.81 ± 0.072	64.43 ± 0.21^##^

Dams were fed CD, LPD or HFD during 4 weeks of pre-pregnancy and pregnancy (CD n=7, LPD n=7, HFD n=7). Birthweight and blood glucose concentration were measured in male newborns within 24 h after birth (n = 7–8 each group). Data represents mean ± SEM. Birth weight - *p < 0,001 CD vs LPD and HFD. BW at weaning - *p < 0,0001 CD vs LPD and HFD. Birth glucose (mg/dL) - p = 0.0098 LPD vs CD and HFD. BW at 10 wks - *p < 0.01 CD-CD vs LDP-CD; **p < 0.01 CD-Cd vs HDF-CD; ^#^p < 0.0001 CD-HFD vs LPD-HFD; ^##^p < 0.0001 CD-HFD vs HFD-HFD. QMR – fat (g) - *p < 0.001 CD-CD vs LPD-CD; **p < 0.01 CD-CD vs HFD-CD; ^##^p < 0.01 CD-HFD vs HFD-HFD. QMR – fat (%) - *p < 0.05 CD-CD vs LPD-CD; **p < 0.001 CD-CD vs HFD-CD; ^#^p < 0.01 CD-HFD vs HFD-HFD. QMR – lean (g) - *p < 0.0001 CD-CD vs LPD-CD; **p < 0.01 CD-CD vs HFD-CD; ^#^p < 0.01 CD-HFD vs LPD-HFD. QMR – lean (%) - **p < 0.001 CD-CD vs HFD-CD; **p < 0.01 CD-CD vs HFD-CD; ^#^p < 0.01 CD-HFD vs HFD-HFD. QMR – water (g) - *p < 0.0001 CD-CD vs LPD-CD; **p < 0.01 CD-CD vs HFD-CD; ^#^p < 0.0001 CD-HFD vs LPD-HFD. QMR – water (%) - **p < 0.001 CD-CD vs HFD-CD; ^##^p < 0.01 CD-HFD vs HFD-HFD.
